# Retraction Note: Decoding intelligence via symmetry and asymmetry

**DOI:** 10.1038/s41598-025-16028-y

**Published:** 2025-08-26

**Authors:** Jianjing Fu, Ching-an Hsiao

**Affiliations:** 1 College of Media Engineering, Communication University of Zhejiang, Hangzhou, China; 2 Fintech Engineering Technology Research Center, Guangdong University of Finance, Guangzhou, China

Retraction of: *Scientific Reports* 10.1038/s41598-024-62906-2, published online 31 May 2024

The Editors have retracted this Article.

After publication, concerns were raised about nonstandard or incoherent language. Specifically, the Article contained many sentences that were irrelevant to the topic and used unusual or inappropriate terms. Post-publication peer review corroborated these concerns, and the Authors’ response was not sufficient to resolve them. The Editors therefore no longer have confidence in the research presented.

The Authors did not respond to the Editors’ correspondence about this retraction.

